# Time-dependent impact of immunosuppressant regimens on cardiovascular outcomes in kidney transplant recipients: a nationwide cohort study

**DOI:** 10.3389/fphar.2025.1540576

**Published:** 2025-05-13

**Authors:** Jinhyun Park, Wonhui Choi, Jinseub Hwang, Young-Mi Ah, Byung Ha Chung, Yun-Kyoung Song

**Affiliations:** ^1^ College of Pharmacy, Daegu Catholic University, Gyeongsan, Republic of Korea; ^2^ Department of Statistics, Daegu University, Gyeongsan, Republic of Korea; ^3^ College of Pharmacy, Yeungnam University, Gyeongsan, Republic of Korea; ^4^ Division of Nephrology, Department of Internal Medicine, Seoul St. Mary’s Hospital, College of Medicine, The Catholic University of Korea, Seoul, Republic of Korea; ^5^ College of Pharmacy, The Catholic University of Korea, Bucheon, Republic of Korea

**Keywords:** kidney transplantation, immunosuppressive agents, cardiovascular outcomes, comorbidity, time-dependent analysis

## Abstract

**Objectives:**

We aimed to evaluate the effect of different immunosuppressive regimens on the risk of major adverse cardiovascular events (MACEs) in kidney transplant recipients (KTRs).

**Methods:**

This retrospective cohort study used nationwide claims data from the Korean Health Insurance Review and Assessment Service from between 2010 and 2021. Immunosuppressive medications were analyzed as time-dependent variables, and the primary outcome was MACEs, defined as a composite of myocardial infarction, coronary revascularization, ischemic stroke, and all-cause mortality.

**Results:**

A total of 8,056 KTRs were included in the analysis, with significant risk factors for MACEs identified as male sex, older age, longer dialysis duration, lower economic status, and greater comorbidity. At the time of the kidney transplant, 86.7% of the KTRs were administered standard triple therapy, after which various immunosuppressive regimens, including sirolimus-inclusive regimens, were employed. The risk of MACE was lower or comparable in KTRs standard triple therapy than in those receiving most other immunosuppressive regimens. However, corticosteroid withdrawal was associated with a significant reduction in cardiovascular risk, particularly in KTRs with preexisting diabetes or dyslipidemia.

**Conclusion:**

These findings suggest that early consideration should be given to minimizing steroid use in KTRs with dyslipidemia or diabetes to optimize cardiovascular outcomes.

## 1 Introduction

With the development of various immunosuppressive agents, the 5-year post-transplant graft survival and patient survival rates have exceeded 75% in kidney transplant recipients (KTRs) (United States Renal Data System, 2023). These rates are particularly high among Asian KTRs, with graft survival rates exceeding 95% in Korea (Lee et al., 2021). Nevertheless, the mortality rate due to cardiovascular disease (CVD) remains higher than that in the general population (Chaudhry et al., 2022; United States Renal Data System, 2023), accounting for approximately 40% of deaths in KTRs (Ying et al., 2020; United States Renal Data System, 2023). Several studies have shown that nonfatal cardiovascular events after kidney transplant (KT) can be linked to increased graft failure and mortality (Lentine et al., 2005; Anderson et al., 2022).

The use of immunosuppressive agents induces metabolic side effects and may be a risk factor for CVD in KTRs (Rangaswami et al., 2019; Aziz et al., 2022). Hypertension and diabetes are affected by the use of calcineurin inhibitors (CNI) and corticosteroids (STR), while dyslipidemia is affected by the use of CNI, STR, and mammalian target of rapamycin (mTOR) inhibitor (Elezaby et al., 2022).

Long-term use of CNI and STR is generally associated with a higher risk of CVD (Chen et al., 2021; Hong et al., 2021; Pofi et al., 2023). However, in KTRs, STR withdrawal and avoidance reportedly do not significantly affect cardiovascular events (Haller et al., 2016), and switching from CNIs or mycophenolic acid to everolimus has no significant impact on the incidence of major adverse cardiovascular events (MACEs) (van Dijk et al., 2018; Sommerer et al., 2023). However, the previous studies examining these effects do not have sufficient follow-up periods to evaluate long-term effects, do not adequately report cardiovascular events, and predominantly involve Caucasian populations.

Racial differences have been observed in cardiovascular risk in the general population and KTRs, as well as in adverse effects from immunosuppressive agents in KTRs (Johnston et al., 2008; Anderson et al., 2022). Although standard triple therapy using CNI, antimetabolites (AM), and STR is commonly used to treat KTRs, the combination of immunosuppressive agents is often adjusted based on an individual patient’s clinical condition. The immunosuppressive regimen can change over time depending on factors such as its clinical effectiveness, adverse drug reactions, infection, and renal function (Kidney Disease: Improving Global Outcomes KDIGO Transplant Work Group, 2009). Consequently, comparisons of the impacts of different drug combinations on MACEs are challenging, and no prior studies have conducted time-dependent analyses on this topic. Therefore, using nationwide claims data, we aimed to evaluate the long-term impact of specific immunosuppressive regimens on MACEs based on their actual use in clinical practice in Korean KTRs.

## 2 Materials and methods

### 2.1 Study design and data source

This study employed a retrospective cohort design, using insurance claims data from between 2010 and 2021, obtained from Korea’s Health Insurance Review and Assessment Service (HIRA). HIRA, an independent public insurance agency, evaluates medical service charges and assesses the necessity of prescribed treatments according to labeling guidelines. Our dataset included anonymized demographic information, diagnostic codes, procedures, and prescription data, with each individual identified using a unique coded identifier (Cheol Seong et al., 2017; Health Insurance Review and Assessment Service, 2022). This study was approved by the Institutional Review Board (IRB) of Daegu Catholic University (IRB No. CUIRB-2022-E009, 24 May 2022), which waived the requirement for informed consent because all patient data were anonymized and de-identified using a randomized identification number before the retrospective analysis.

### 2.2 Study population

We included all KTRs treated between 2011 and 2020 who were identified by the procedure code for KT (R3280) and the hospitalization code (Kang et al., 2022). The index date was defined as the date of KT. The exclusion criteria were as follows: 1) KTRs under 20 years of age, 2) who were deceased by the index date, 3) with a history of kidney transplantation before the index date, 4) who had received non-kidney transplants before the KT date or subsequently, 5) who were diagnosed with cancer (International Classification of Diseases, 10th Revision [ICD-10] codes: C00−C99) during the study period, 6) had a history of being diagnosed with ischemic heart disease (ICD10 codes: I20−I25), valve disorders (ICD-10 codes: I34−I36), stroke (ICD-10 codes: I60−I69) or MACEs within 1 year prior to the KT date, and 7) who experienced acute rejection. Given the limitations of the Korea National Health Insurance Service claims data in specifying the exact timing of kidney allograft rejection, acute rejection was defined as a diagnosis of kidney allograft rejection, identified by ICD-10 codes T86 and/or T86.1, recorded within 3 months post-transplant (Lee et al., 2021).

### 2.3 Exposure

**FIGURE 1:**
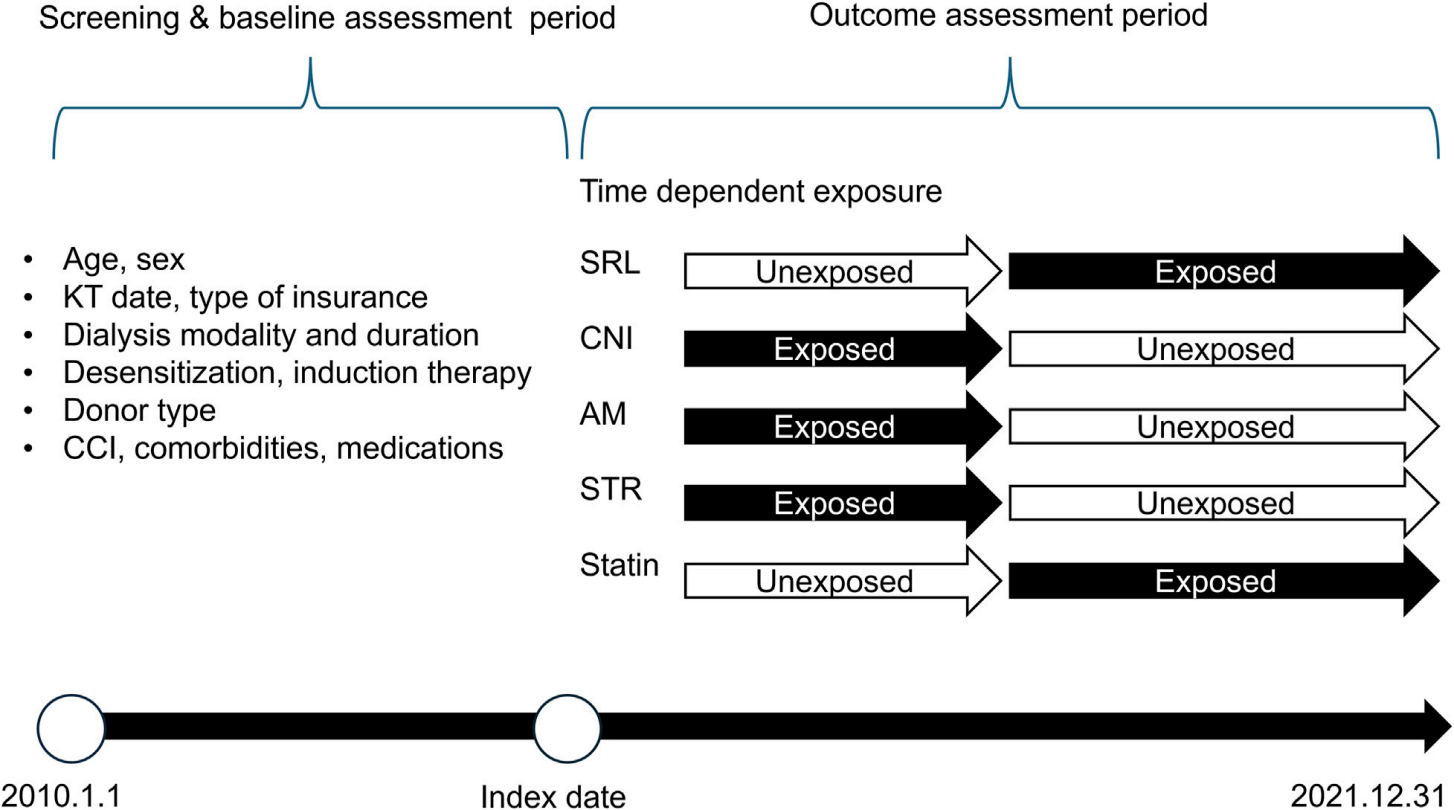
Study timeline and design. Dialysis duration and modality were analyzed using all available data prior to the Index date Abbreviation: KT, kidney transplant; CCI, charlson comorbidity index; SRL, sirolimus; CNI, calcineurin inhibitor; AM, antimetabolite; STR, corticosteroid; statin, 3-hydroxy-3-methylglutaryl coenzyme A reductase inhibitors.

We classified the immunosuppressive agents into four categories (Figure 1): CNIs, including cyclosporine and tacrolimus; mTOR inhibitors; AM; and STR. Owing to reimbursement issues in Korea, the mTOR inhibitor category included only sirolimus (SRL) (Ministry of Health and Welfare, 2022). The exposure period was determined using the initiation date of each prescription and cumulative days of administration obtained from the claims data. Exposure to immunosuppressive agents was considered a time-dependent variable. We did not extend the exposure period when there was an overlap in exposure periods for the same class of immunosuppressive agents.

### 2.4 Outcomes

The primary outcome of the study was the occurrence of MACEs, which were defined as composite outcomes identified through ICD-10 codes for myocardial infarction, coronary revascularization, ischemic stroke, and all-cause mortality. Myocardial infarction was determined using ICD-10 codes I21–22 in conjunction with hospitalization. Coronary revascularization was performed using specific procedure codes for angioplasty or bypass surgery (codes: M6551–M6554, M6561–M6567, M6571, M6572, M6620, M6634, M6638, O1640–O1642, O1647–O1649, OA640–OA642, OA647–OA649). Ischemic stroke was defined using the ICD-10 code I63 with hospitalization. All-cause mortality was identified based on claims that indicated death as the medical outcome. The date of the first occurrence of any of these events was recorded as the composite cardiovascular outcome.

Secondary outcomes included the individual components of MACEs and death-censored graft failure (DCGF). DCGF was defined as the need for consecutive dialysis sessions lasting >90 days post-transplantation, allograft nephrectomy (procedure code: R3275), or retransplantation. The outcome date was marked by the initiation of dialysis or date of the relevant procedure. Follow-up was terminated upon the occurrence of DCGF or MACEs or at the conclusion of the study.

### 2.5 Covariates

The baseline characteristics were assessed prior to the index date. These included factors such as age, sex, index year, insurance type, and dialysis-related details such as the duration and modality of dialysis. Other variables included the type of kidney donor, whether the KTRs underwent desensitization or induction therapy, the Charlson comorbidity index (CCI) score, and key comorbidities such as dyslipidemia, diabetes, and hypertension. Medications for CVD, including statins, anticoagulants, and antiplatelet agents, were also considered (Figure 1; Supplementary Table S1).

The duration of dialysis was measured from the start of dialysis to the date of KT, with preemptive KT classified for those who had undergone <3 months of dialysis (Kang et al., 2022). Hemodialysis was defined by procedure codes (O7020, O9992, O9993, OH011), and peritoneal dialysis by procedure codes (O7061, O7062, O7071-7) with claims records for peritoneal dialysis solution (Choi et al., 2014).

In South Korea, living-donor KT recipients bear the cost of donor nephrectomy, whereas deceased-donor organ donations are government-funded. Therefore, if the procedure code for donor nephrectomy (R3272) was billed to the recipient, the donor type was classified as living donor (Lee et al., 2021). To assess the immunological risk in KTRs, data on desensitization and induction therapy were collected. Desensitization was defined as the use of rituximab, intravenous immunoglobulin, or plasmapheresis (procedure code: X2505) (Lee and Kang, 2015; Park et al., 2021). Induction regimens such as basiliximab and rabbit anti-thymocyte globulin (rATG) were also extracted. The CCI score was based on ICD-10 codes from 1 year before the transplant, with ESRD patients having a minimum score of 2 (Quan et al., 2005). The post-KT use of statins was expected to have a significant impact on the outcomes and was therefore considered a time-dependent variable.

### 2.6 Statistical analysis

Categorical variables were summarized as frequencies and percentages, and continuous data as means with standard deviations. For each immunosuppressant regimen period, the incidence rates were calculated as the number of events divided by the corresponding person-time. To assess the influence of specific immunosuppressive regimens on MACEs, we conducted a time-dependent Cox proportional hazards analysis. The analysis included covariates such as age, sex, index year, type of insurance, dialysis duration, donor type, desensitization, induction therapy, CCI score, comorbidities including dyslipidemia, diabetes mellitus, and hypertension, and cardiovascular medications (statins, anticoagulants, and antiplatelet agents). Additionally, we evaluated the interaction effects of combinations of immunosuppressive agents. Hazard ratios (HR) for specific immunosuppressive regimens were compared with those for standard triple therapy, which consisted of CNI, AM, and STR.

Furthermore, we analyzed the influence of specific immunosuppressive regimens based on the underlying diseases, including diabetes and dyslipidemia. To test the robustness of our findings, prescription data from 1-year post-KT were analyzed. All statistical analyses were performed using SAS Enterprise Guide 7.1 (The SAS Institute, Cary, NC, USA) or R (version 3.5.1; R Foundation for Statistical Computing, Vienna, Austria; http://www.R-project.org/) software. Statistical significance was set at a two-sided p < 0.05.

## 3 Results

### 3.1 Baseline characteristics

**FIGURE 2:**
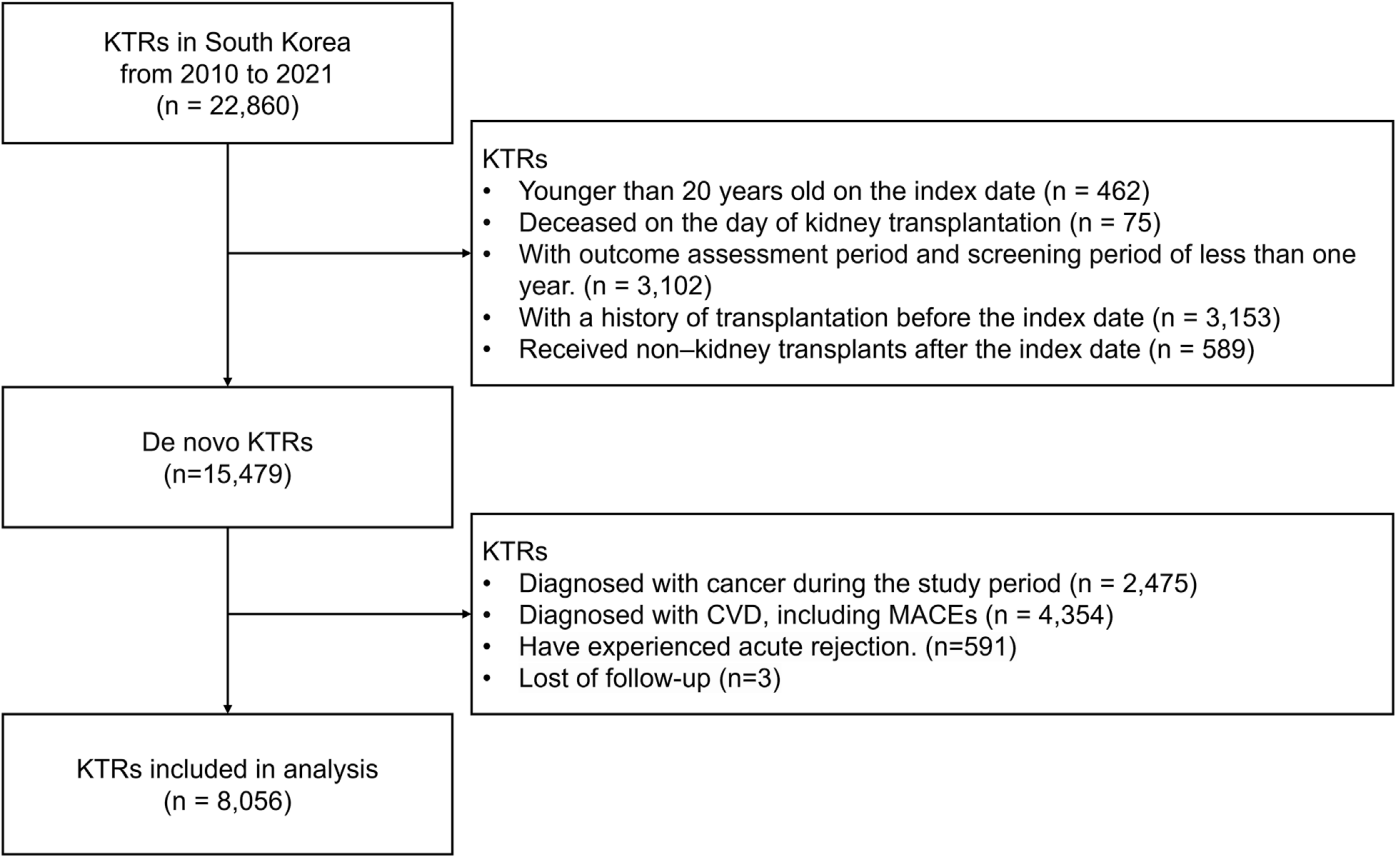
Study cohort selection process. Abbreviation: KTRs, kidney transplant recipients; CVD, cardiovascular disease; MACE, major adverse cardiovascular events; mTOR, mammalian target of rapamycin.

Among the 22,860 KTRs treated between 2010 and 2021, 14,804 were excluded based on the predefined exclusion criteria. The eligible study cohort included 8,056 KTRs (Figure 2).

The baseline characteristics of the KTRs included in this analysis are listed in Table 1. Of the 8,056 KTRs, 58.27% were male (mean age: 46.75 years). The proportions of KTRs aged ≤40 years, in their 40s, and in their 50s were similar; however, those aged ≥60 years accounted for 13.18%, which was lower than the percentages in the other age groups. As the years progressed, the number of KTRs performed each year increased. Most KTRs were covered by health insurance (88.01%) and 35.17% were preemptive transplant recipients. Approximately 45.12% of the KTRs underwent hemodialysis prior to KT, whereas 10.92% underwent peritoneal dialysis. The deceased donor transplantation rate was 20.38%, which was lower than that for living-donor transplantation (79.62%). Desensitization and induction therapies were administered to 18.63% and 98.29% of KTRs, respectively, with 18.47% of KTRs receiving rATG for induction therapy. A CCI score of three was most prevalent among KTRs (23.63%). Most KTRs had hypertension (88.70%) and dyslipidemia (69.91%) before KT, whereas the prevalence of diabetes was 42.96%. Additionally, 57.98% of the KTRs were taking statins prior to KT, and 45.43% were on anticoagulant therapy.

**TABLE 1:** Baseline characteristics of kidney transplant recipients included in the analysis.

Characteristics	KTRs included in the analysis (N = 8,056)
Age (years, mean ± SD)	46.75 (11.34)
<40	2,185 (27.12)
40–49	2,293 (28.46)
50–59	2,516 (31.23)
60≤	1,062 (13.18)
Sex	
Male	4,694 (58.27)
Female	3,362 (41.73)
Kidney transplant year	
2011	658 (8.17)
2012	699 (8.68)
2013	697 (8.65)
2014	700 (8.69)
2015	766 (9.51)
2016	910 (11.30)
2017	909 (11.28)
2018	856 (10.63)
2019	918 (11.40)
2020	943 (11.71)
Type of insurance	
Health insurance	7,090 (88.01)
Medical aid	966 (11.99)
Dialysis duration (year)	
Preemptive (<0.25)	2,821 (35.02)
0.25–1.5	1,581 (19.63)
1.5–3.0	1,127 (13.99)
3.0≤	2,527 (31.37)
Dialysis modality	
Hemodialysis	3,635 (45.12)
Peritoneal dialysis	880 (10.92)
Mixed^a^	720 (8.94)
Preemptive	2,821 (35.02)
Donor type	
Deceased donor	1,642 (20.38)
Living donor	6,414 (79.62)
Desensitization	1,501 (18.63)
Plasmapheresis	1,246 (15.47)
Rituximab	1,343 (16.67)
Intravenous immunoglobulin^b^	70 (0.87)
Induction therapy	7,918 (98.29)
Basiliximab	6,629 (82.29)
rATG	1,488 (18.47)
Charlson comorbidity index score	
2	1,759 (21.83)
3	1,904 (23.63)
4	1,488 (18.47)
5	1,208 (15.00)
6≤	1,697 (21.07)
Underlying disease	
Hypertension	7,146 (88.70)
Diabetes	3,461 (42.96)
Dyslipidemia	5,632 (69.91)
Congestive heart failure	1,297 (16.10)
Atrial fibrillation	143 (1.78)
Co-medication	
Statin	4,671 (57.98)
Anticoagulant	3,660 (45.43)
Antiplatelet agent	157 (1.95)

Data are shown as number of kidney transplant recipients (percentage).

^a^ KTRs, who used both hemodialysis and peritoneal dialysis.

^b^ The use of intravenous immunoglobulin for desensitization is not reimbursed in Korea and is therefore not reflected in the HIRA, database. (Ministry of Health and Welfare, 2021).

Abbreviations: KTR, kidney transplant recipients; SD, standard deviation.

### 3.2 Risk factors for MACEs

The risk factors for MACEs are presented in Table 2, based on the results of the multivariate Cox regression analysis. Factors associated with a reduced risk of MACE included female sex (HR: 0.57, 95% confidence interval [CI]: 0.47–0.71) and post-transplant statin use (HR: 0.70, 95% CI: 0.57–0.86).

**TABLE 2:** Risk factors associated with MACE.

Variable	Events, n (%)	Multivariate analysis	
		aHR (95%CI)	p-value
Age			
<40	43 (1.97)	Reference	
40–49	93 (4.06)	2.13 (1.48–3.06)	<0.0001
50–59	194 (7.71)	4.14 (2.95–5.80)	<0.0001
60≤	129 (12.15)	7.67 (5.33–11.02)	<0.0001
Sex			
Male	329 (7.01)	Reference	
Female	130 (3.87)	0.57 (0.47–0.71)	<0.0001
Kidney transplant year			
2011	54 (11.76)	Reference	
2012	54 (7.73)	0.87 (0.58–1.29)	0.4801
2013	62 (8.9)	1.08 (0.72–1.62)	0.6948
2014	54 (7.71)	1.05 (0.68–1.61)	0.8225
2015	49 (6.4)	1.00 (0.64–1.56)	0.9988
2016	54 (5.93)	0.95 (0.61–1.45)	0.8315
2017	43 (4.73)	0.96 (0.60–1.53)	0.846
2018	36 (4.21)	1.09 (0.67–1.78)	0.7294
2019	24 (2.61)	0.81 (0.47–1.41)	0.4563
2020	29 (3.08)	1.40 (0.81–2.40)	0.2294
Type of insurance			
Health insurance	372 (5.25)	Reference	
Medical aid	87 (9.01)	1.96 (1.53–2.52)	<0.0001
Dialysis duration			
Preemptive (<0.25)	101 (3.58)	Reference	
0.25–1.5	82 (5.19)	1.23 (0.91–1.66)	0.1793
1.5–3.0	92 (8.16)	1.60 (1.18–2.16)	0.0024
3.0≤	184 (7.28)	1.76 (1.35–2.31)	<0.0001
Donor type			
Deceased donor	136 (8.28)	Reference	
Living donor	323 (5.04)	0.92 (0.74–1.14)	0.4444
Desensitization	65 (4.33)	1.06 (0.81–1.40)	0.6626
Induction therapy			
Basiliximab	383 (5.78)	1.35 (0.86–2.12)	0.1965
rATG	88 (5.91)	1.29 (0.84–1.99)	0.2495
CCI score			
2	66 (3.75)	Reference	
3	90 (4.73)	1.28 (0.93–1.76)	0.1363
4	73 (4.91)	1.21 (0.86–1.70)	0.2675
5	81 (6.71)	1.48 (1.06–2.08)	0.022
6≤	149 (8.78)	1.95 (1.43–2.65)	<0.0001
Underlying disease			
Hypertension	405 (5.67)	1.09 (0.81–1.48)	0.5633
Dyslipidemia	311 (5.52)	1.08 (0.87–1.35)	0.4728
Arterial fibrillation	25 (17.48)	2.47 (1.64–3.73)	<0.0001
Post-KT statin^a^	N/A	0.68 (0.58, 0.80)	<0.0001

^a^ Statin use after kidney transplant was analyzed as a time-dependent variable in the Cox regression model.

Abbreviations: MACE, major adverse cardiovascular event; aHR, adjusted hazard ratio; CI, confidence interval; HR, hazard ratio; rATG, rabbit anti thymocyte globulin; KT, kidney transplant; N/A, not applicable.

Conversely, factors associated with a higher risk of MACEs included older age (<40 years: HR 2.13, 95% CI: 1.48–3.06; 50–59 years: HR: 4.14, 95% CI: 2.95–5.80; ≥60 years: HR: 7.67, 95% CI: 5.33–11.02), longer dialysis duration before transplantation (1.5–3 years: HR: 1.60, 95% CI: 1.18–2.16; >3 years: HR: 1.76, 95% CI: 1.35–2.31), lower economic status (HR: 1.96, 95% CI: 1.53–2.52), a higher CCI score (5 HR: 1.48, 95% CI: 1.06–2.08; 6 or higher: HR: 1.95, 95% CI: 1.43–2.65), and the presence of atrial fibrillation before KT (HR: 2.47, 95% CI: 1.64–3.73).

### 3.3 Impact of immunosuppressive regimens on MACE and secondary outcomes over time

**FIGURE 3:**
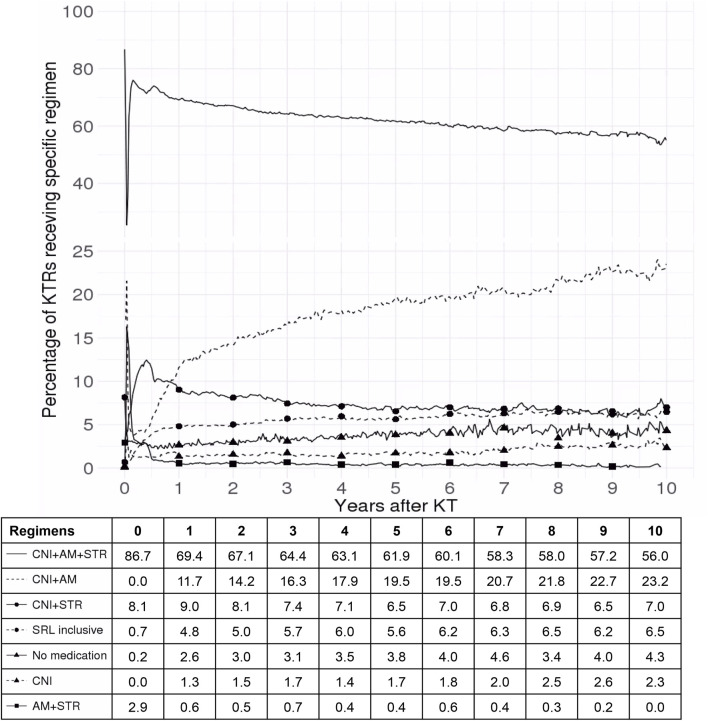
Trends in immunosuppressive regimen use among kidney transplant recipients over 10 years. This figure illustrates the percentage of kidney transplant recipients receiving various immunosuppressive regimens over a 10-year period following kidney transplant. The solid line represents CNI + AM + STR, the dashed line indicates CNI + AM, the solid line with circular markers shows CNI + STR, the dashed line with circular markers denotes SRL-inclusive regimens, the solid line with triangular markers reflects patients on no medication, the dashed line with triangular markers represents CNI alone, and the solid line with square markers depicts AM + STR. The x-axis represents the number of years after kidney transplant and the y-axis indicates the percentage of kidney transplant recipients on each specific regimen. Abbreviations: SRL, sirolimus; CNI, calcineurin inhibitor; AM, antimetabolite; STR, corticosteroid; KT, kidney transplant; KTR, kidney transplant recipient.

Figure 3 demonstrates the substantial changes in the immunosuppressive regimens during the first year after KT. A marked increase in the use of SRL-inclusive regimens was observed as well as a significant rise in STR withdrawal throughout this period. Subsequently, the proportion of KTRs receiving standard triple therapy gradually decreased (from 86.7% to 56.0%).

In the analysis of MACEs, 8,056 KTRs provided 43,226 person-years of follow-up data. The mean follow-up duration was 5.36 years, with an incidence rate of MACE observed at 10.6 cases per 1,000 person-years. Of the 459 MACEs identified, death accounted for the largest proportion (n = 162), followed by ischemic stroke (n = 144), revascularization (n = 80), and myocardial infarction (n = 73). The cumulative incidences of MACE within 1 and 3 years after KT were 1.66% and 3.51%, respectively (Supplementary Figure S1).

**FIGURE 4:**
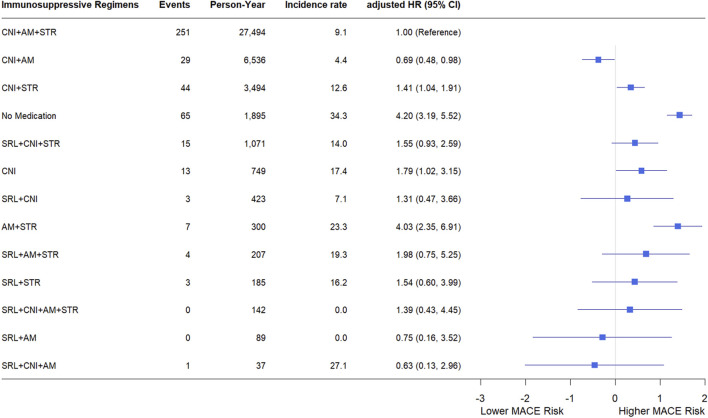
Incidence rate and hazard ratio of MACE according to immunosuppressive regimens. Each immunosuppressive regimen is represented by four types of immunosuppressive medication (SRL, CNI, AM, STR). incidence rates are expressed as the number of events per 1,000 person-years. Hazard ratios were adjusted for sex, age, transplant year, type of insurance, dialysis duration, donor type, presence or absence of desensitization, drugs used in induction therapy, CCI, underlying disease. Forest plot illustrating hazard ratios for major adverse cardiovascular events. The x-axis is presented on a natural log scale. Horizontal lines represent the 95% confidence intervals. Abbreviations: SRL, sirolimus; CNI, calcineurin inhibitor; AM, antimetabolite; STR, corticosteroid; PY, person-year; IR, incidence rate; HR, hazard ratio; CI, confidence interval.

Figure 4 shows the incidence rates and HRs of MACE according to the immunosuppressive regimens. Of the total person-years, 63.61% consisted of standard triple therapy (CNI, AM, and STR). During the 27,494 person-years of standard triple therapy, 251 MACEs occurred, with an incidence rate of 9.1 cases per 1,000 person-years. During the 6,536 person-years of the regimen which included CNI and AM, 29 MACEs were diagnosed, with an incidence rate of 4.4 cases per 1,000 person-years. The withdrawal of STR can reduce the risk of MACE. Regimens that had a negative impact on MACE compared with triple therapy were CNI + STR, no medication, CNI, and AM + STR. None of the other regimens affected the risk of MACE compared with triple therapy. The risk of MACE at 1 year post-KT was consistent with that for the entire study period. Detailed information is provided in Supplementary Figure S2.

The incidence rates and HRs for the secondary outcomes are shown in Supplementary Figure S3. Most of the results were similar to those observed for MACE. However, for STR withdrawal, no significant differences in other outcomes were observed compared with triple therapy, except for stroke. Additionally, when CNI was replaced with SRL, the all-cause mortality increased significantly. Conversely, the addition of SRL to triple therapy resulted in favorable outcome for DCGF.

### 3.4 Subgroup analysis according to comorbidity based on immunosuppressive regimens

**FIGURE 5:**
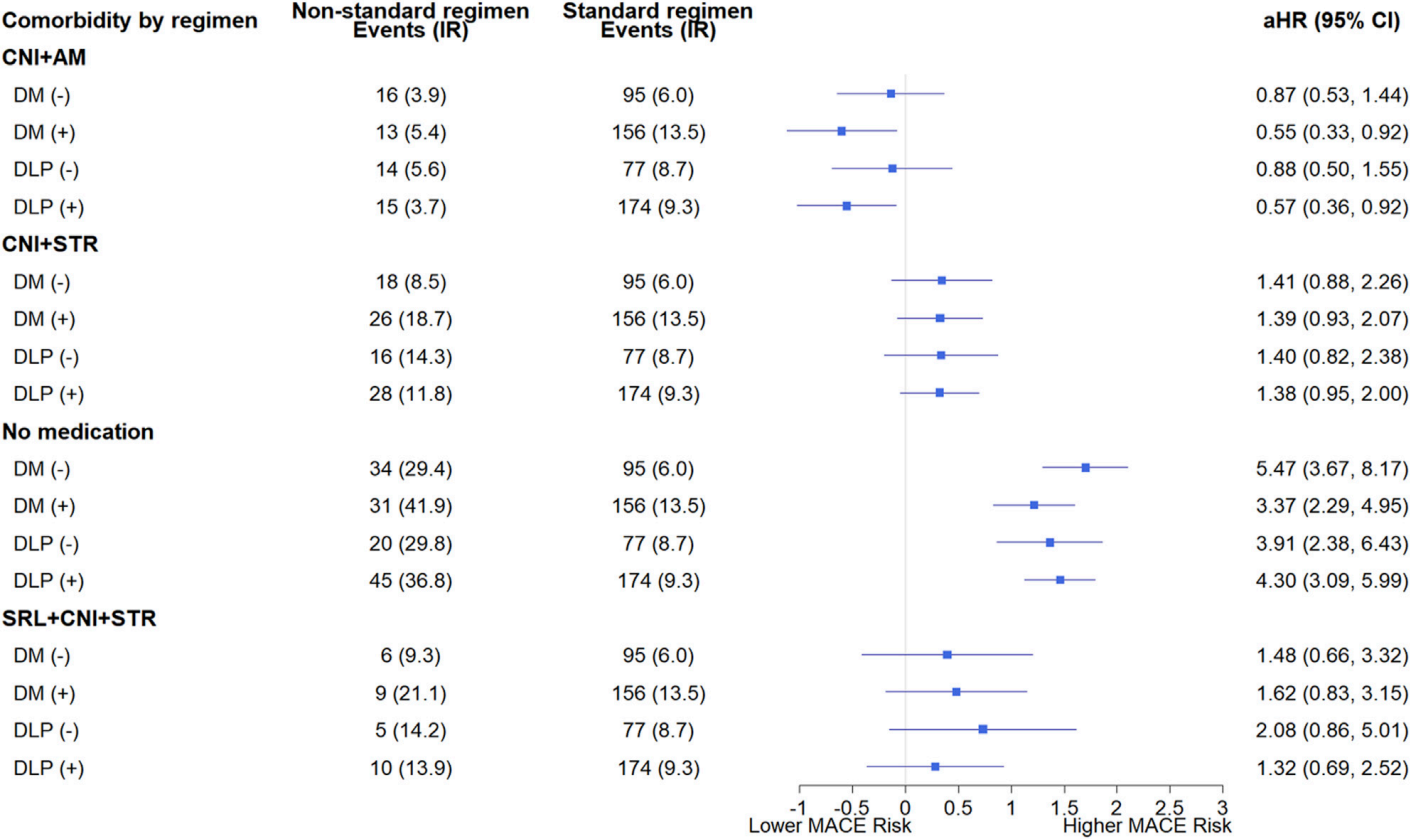
Subgroup analysis of major adverse cardiovascular events based on the presence or absence of pre-transplant diabetes and dyslipidemia according to immunosuppressive regimens. Each immunosuppressive regimen is represented by four types of immunosuppressive medication (SRL, CNI, AM, STR). Hazard ratios were adjusted for sex, age, transplant year, type of insurance, dialysis duration, donor type, presence or absence of desensitization, drugs used in induction therapy, CCI, underlying disease. Forest plot illustrating hazard ratios for major adverse cardiovascular events. The x-axis is presented on a natural log scale. Horizontal lines represent the 95% confidence intervals. (−) indicates the absence, and (+) indicates the presence of comorbidities. The standard regimen refers to triple therapy consisting of CNI, AM, and STR. Abbreviations: DM, diabetes; DLP, dyslipidemia; SRL, sirolimus; CNI, calcineurin inhibitor; AM, antimetabolite; STR, corticosteroid; aHR, adjusted hazard ratio; CI, confidence interval.

The results of the subgroup analyses according to comorbidity (diabetes and dyslipidemia) are presented in Figure 5. The HRs for the immunosuppressive regimens compared with standard triple therapy varied depending on the presence of diabetes and dyslipidemia. In KTRs with diabetes and dyslipidemia, the withdrawal of STR was associated with a reduction in MACE, with HRs of 0.55 (95% CI: 0.33–0.92) and 0.57 (95% CI: 0.36–0.92), respectively. In contrast, no significant reduction was observed in KTRs without this condition.

## 4 Discussion

To our knowledge, this study is the first to conduct a long-term, time-dependent analysis of the effects of immunosuppressive agents on Asian KTRs. Furthermore, this study investigated time-dependent changes in immunosuppressive agent use following KT, and assessed the manner in which these adjustments influence long-term cardiovascular outcomes. Standard triple therapy, including CNI, AM, and STR, was either superior to or not significantly different from most other regimens in terms of cardiovascular risk, except for STR withdrawal. STR withdrawal significantly reduced cardiovascular risk, especially in KTRs with preexisting diabetes or dyslipidemia.

Stable maintenance immunosuppression is typically established within the first 3 months following KT in most KTRs. While some centers may continue to taper immunosuppressive therapy up to 1-year post-transplant, modifications to the initial regimen may be necessary in response to post-transplant complications such as toxicity, graft dysfunction, acute rejection, infection, or malignancy (Kidney Disease: Improving Global Outcomes KDIGO Transplant Work Group, 2009; Hardinger and Brennan, 2020). In Korean KTRs, 1 year post-transplant, maintenance regimens have been reported to primarily consist of standard triple therapy (67.5%), CNI+AM (13.2%), CNI+STR (6.5%), and SRL-containing regimens (7.5%), reflecting trends similar to those observed in current study. The most common modification in therapy was the discontinuation of AM, primarily due to gastrointestinal complications and infections (Chang et al., 2017).

The overall incidence of MACE in this study was lower than that previously reported. Earlier studies have documented an annual cardiovascular event rate of 3.5%–5% in KTRs (Kidney Disease: Improving Global Outcomes KDIGO Transplant Work Group, 2009), with myocardial infarction rates of 5.6% at 1 year and 11.1% at 3 years post-KT (Lentine et al., 2005). Furthermore, Within 1 year post-KT, the hospitalization rate due to CVD was 120 cases per 1,000 person-years (United States Renal Data System, 2023). The lower incidence of MACE in our cohort may be attributed to the characteristics of the Asian population, which differs from Western populations in cardiovascular risk factors. It may also be associated with the specific characteristics of the Korean KT cohort, which had a high proportion of living-donor transplants (Okumi et al., 2018). Additionally, the exclusion of KTRs with a history of cancer or prior cardiovascular events likely reduced the presence of high-risk KTR. The other baseline characteristics were similar to those observed in Korean KTRs (Park et al., 2020).

STR has long been used because of its anti-inflammatory and immunosuppressive properties. Although effective in reducing acute rejection, STR is associated with various adverse effects, particularly its potential to induce hypertension, hyperglycemia, and dyslipidemia, which adversely affect cardiovascular and metabolic outcomes (Pofi et al., 2023).

In this study, the regimen that excluded STR showed a more positive effect on MACE than triple therapy (HR 0.69, 95% CI: 0.48–0.98). The risk for stroke was also reduced significantly, but no statistically significant differences were observed for other secondary outcomes. STR withdrawal and avoidance are associated with a relatively reduced risk of cardiovascular event up to 5 years, with the risk ratios (RR) of 0.98 (95% CI: 0.42–2.33) and 0.56 (95% CI: 0.30–1.05), respectively, compared with STR maintenance (Haller et al., 2016). The use of STR therapy in patients with preexisting diabetes is associated with worsening of glycemic control. When patients are administered a daily dose equivalent to 7.5 mg or more of prednisolone, there is an increase in the risk of cardiovascular events (Wei et al., 2004). The current study found that STR use negatively impacted MACE in KTRs with preexisting diabetes and dyslipidemia, which is consistent with the findings of previous studies In the current study, the regimen that excluded STR did not affect all-cause death compared with standard triple therapy (HR 0.65, 95% CI: 0.34–1.26). Haller et al. (Haller et al., 2016) also showed that the risk of 1-year mortality for STR withdrawal and avoidance was not significantly different with the triple regimen with the RR (95% CI) of 0.68 (95% CI: 0.36–1.30) and 0.96 (95% CI: 0.52–1.80), respectively. Furthermore, the current study also found no impact of STR withdrawal on DCGF compared with triple therapy (HR 1.14, 95% CI: 0.68–1.89), which was similar results with the previous study (Haller et al., 2016).

CNIs are essential for the prevention of acute and chronic rejections in KTRs. However, they are associated with significant nephrotoxicity, increased susceptibility to infection, and malignancy. CNI also contribute to adverse effects such as cardiac hypertrophy, hypertension, vascular remodeling, and dyslipidemia (Elezaby et al., 2022). To mitigate these complications, various strategies are employed post-KT, including CNI replacement with SRL, CNI discontinuation, and CNI minimization combined with SRL. In the current study, replacement of the CNIs with SRL in triple therapy did not have a significant impact on MACE and DCGF. However, mortality significantly increased (HR 6.74, 95% CI: 2.25–20.15). According to previous studies, the replacement of CNIs with mTOR inhibitors also resulted in an insignificant difference in the risk of mortality and graft loss [RR (95% CI), 0.99 (0.96–1.40) and 0.94 (0.75–1.19), respectively], but an increased risk of hyperlipidemia with an RR of 1.76 (95% CI: 1.4–2.2) (Karpe et al., 2017).

In the current study, compared with standard triple therapy, CNI withdrawal had a negative impact on MACE, all-cause death, and DCGF. However, in the Cochrane review, CNI withdrawal resulted in a mortality RR of 1.09 (95% CI: 0.96–1.24), and graft loss RR of 0.85 (95% CI: 0.74–0.98) (Karpe et al., 2017). As shown in Figure 3, CNI withdrawal frequently occurred within the first year post-transplant, likely due to delayed graft function. Unlike in RCTs, CNI discontinuation in real-world settings often results from various adverse effects (Chang et al., 2017; Hardinger and Brennan, 2020), potentially impacting patient mortality.

AM reportedly do not cause metabolic disorders that lead to increased cardiovascular risk (Elezaby et al., 2022). In the current study, the removal of AM from the triple therapy regimen resulted in a significant negative impact on MACE (HR 1.41, 95% CI: 1.04–1.91). In clinical practice, AM is most frequently discontinued due to adverse effects such as infections and bone marrow suppression, which supports the findings of this study (Chang et al., 2017). When AM was replaced with SRL, no significant effect on MACE was observed (HR 1.31, 95% CI: 0.80–2.13). Similarly, the substitution of AM with another mTOR inhibitor, everolimus, was reported not to affect MACE significantly (RR 0.91, 95% CI: 0.68–1.21) (Sommerer et al., 2023).

As in previous studies, sex, age, dialysis duration, desensitization, CCI, atrial fibrillation, and post-KT statin use were identified as significant risk factors for MACE (Rao and Coates, 2018; Devine et al., 2019; Wyld et al., 2021; Anderson et al., 2022; Sommerer et al., 2023). Hypertension, and dyslipidemia were expected to be potential risk factors before KT. However, they did not significantly increase the risk of MACE in this study. This can be attributed to the development of these conditions in certain KTRs during the long-term follow-up, even if they were free of them before the KT (Kang et al., 2022). Additionally, the effect of these risk factors was likely mitigated as KTRs continuously monitored and managed them in accordance with the established guidelines. (KDIGO clinical practice guideline for the care of kidney transplant recipients., 2009).

This study had a few limitations. The use of standardized immunosuppressive regimens accounted for >50% of the total person-time, and the relatively low incidence of MACE may have contributed to the significant variability in the HRs for minor immunosuppressive regimens, potentially leading to a lack of statistical significance. Nevertheless, with an overall event-per-variable >10, the results for clinically meaningful major immunosuppressive regimens demonstrated sufficient statistical power (Vittinghoff and McCulloch, 2007).

Owing to the limitations inherent in the claims data, certain details were unavailable. Variables such as smoking status, obesity, and kidney function, which may have acted as confounding factors, could not be examined. In South Korea, while approximately 8.6% of KTRs continued smoking after the procedure (Jeon et al., 2022; Jeong et al., 2022), 43.1% of these KTRs quit smoking within 1 year post-KT (Jung et al., 2021). Smoking history showed no significant impact on CVD risk in Korean KTRs (Jeong et al., 2010), with only current smoking after transplantation posing a CVD risk (Weinrauch et al., 2018). We analyzed the occurrence of DCGF as a secondary endpoint owing to the unavailability of kidney allograft function parameters, including urine volume, urine protein excretion, and serum creatinine. In addition, high-risk KTRs with a history of cancer or multi-organ transplantation (i.e., simultaneous pancreas-kidney transplant recipients) were excluded from the analysis to minimize potential confounding factors unrelated to immunosuppressive regimens. While this approach may limit the generalizability of our findings to all kidney transplant recipients, it enabled a more homogeneous study population and allowed for a clearer assessment of the relationship between immunosuppressive regimens and MACE risk in KTRs. Despite these limitations, HIRA data provides a comprehensive dataset representing the entire Korean population, facilitating the analysis of extended trends over time owing to its long-term accumulation. Additionally, it reflects real-world clinical practice, making it valuable for studies based on routine clinical data.

This study presents the first long-term time-dependent analysis of the effects of immunosuppressive regimens on MACE in Korean KTRs. The results demonstrate that the risk of MACE was lower or comparable in KTRs standard triple therapy than in those receiving most other immunosuppressive regimens. However, STR withdrawal leads to a notable reduction in the cardiovascular risk, especially in KTRs with preexisting diabetes or dyslipidemia. These findings suggest that early consideration should be given to minimizing steroid use in KTRs with dyslipidemia or diabetes to optimize cardiovascular outcomes.

## Data Availability

The data analyzed in this study is subject to the following licenses/restrictions: Data may be obtained from a third party and are not publicly available. The data that support the findings of this study are available from the Health Insurance Review and Assessment (HIRA), but restrictions apply to the availability of these data, which were used under license for the current study, and so are not publicly available (https://opendata.hira.or.kr/). Requests to access these datasets should be directed to https://opendata.hira.or.kr/.
