# Supplementary material for: Time-dependent impact of immunosuppressant regimens on cardiovascular outcomes in kidney transplant recipients: a nationwide cohort study
Source: Front Pharmacol. 2025 May 13;16:1540576. doi: 10.3389/fphar.2025.1540576 (PMC12106356; doi:10.3389/fphar.2025.1540576)
Supplement: Supplementary file 1 [file DataSheet1.docx]

Supplementary Material

**Supplementary Table 1. List of co-medications included in the analysis by drug classes**

| Drug class | Medications |
| --- | --- |
| Statins | Atorvastatin, fluvastatin, lovastatin, pitavastatin, pravastatin, rosuvasatin, simvasatin |
| Anticoagulants | Apixaban, dabigatran, edoxaban, rivaroxaban, warfarin |
| Antiplatelet agents | Aspirin, cilostazol, clopidogrel, prasugrel, ticagrelor, ticlopidine, triflusal |


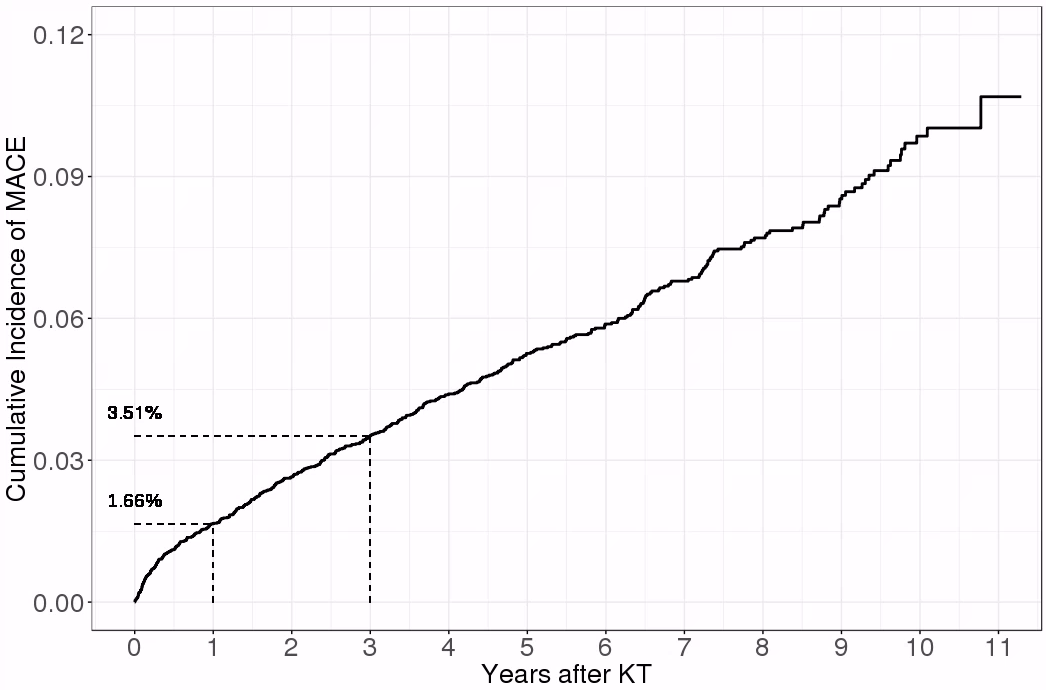


**Supplementary Figure 1 Cumulative incidence of major adverse cardiovascular events over time after KT**

The solid line shows the cumulative incidence of MACE over time, with observed values of 1.66% at 1 year and 3.51% at 3 years post-KT. The x-axis indicates the number of years since KT, and the y-axis represents the cumulative incidence of MACE.

Abbreviations: MACE, major adverse cardiovascular event; KT, kidney transplant.


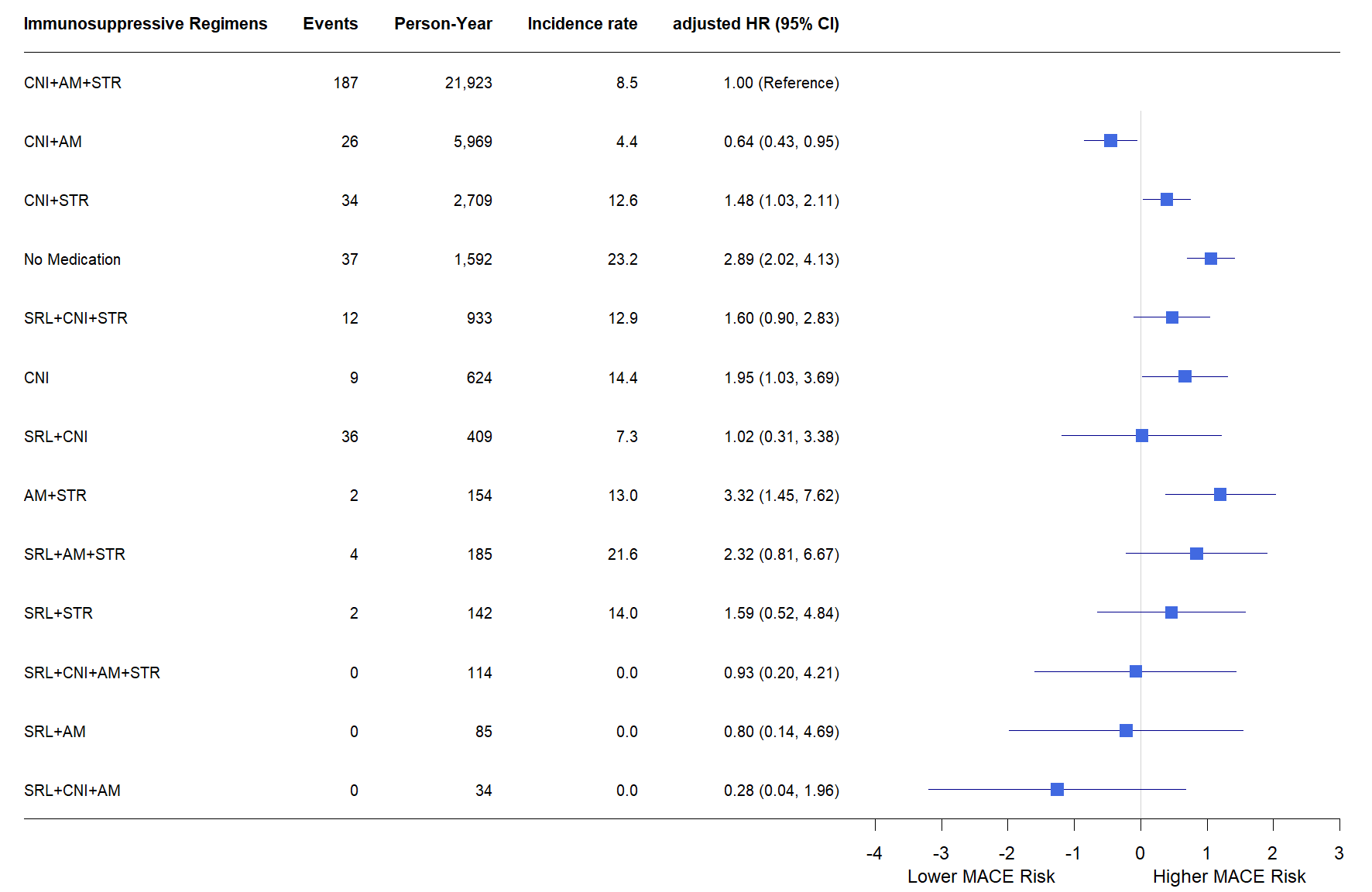


**Supplementary Figure 2 Sensitivity analysis of incidence rate and hazard ratio of MACE within one-year post-KT according to immunosuppressive regimens**

Each immunosuppressive regimen is represented by four types of immunosuppressive medication (SRL, CNI, AM, STR). incidence rates are expressed as the number of events per 1,000 person-years. Hazard ratios were adjusted for sex, age, transplant year, type of insurance, dialysis duration, donor type, presence or absence of desensitization, drugs used in Induction Therapy, CCI, underlying disease. Forest plot illustrating hazard ratios for major adverse cardiovascular events. The x-axis is presented on a natural log scale. Horizontal lines represent the 95% confidence intervals.

Abbreviations: SRL, sirolimus; CNI, calcineurin inhibitor; AM, antimetabolite; STR, corticosteroid; PY, person-year; IR, incidence rate; HR, hazard ratio; CI, confidence interval.

(A)


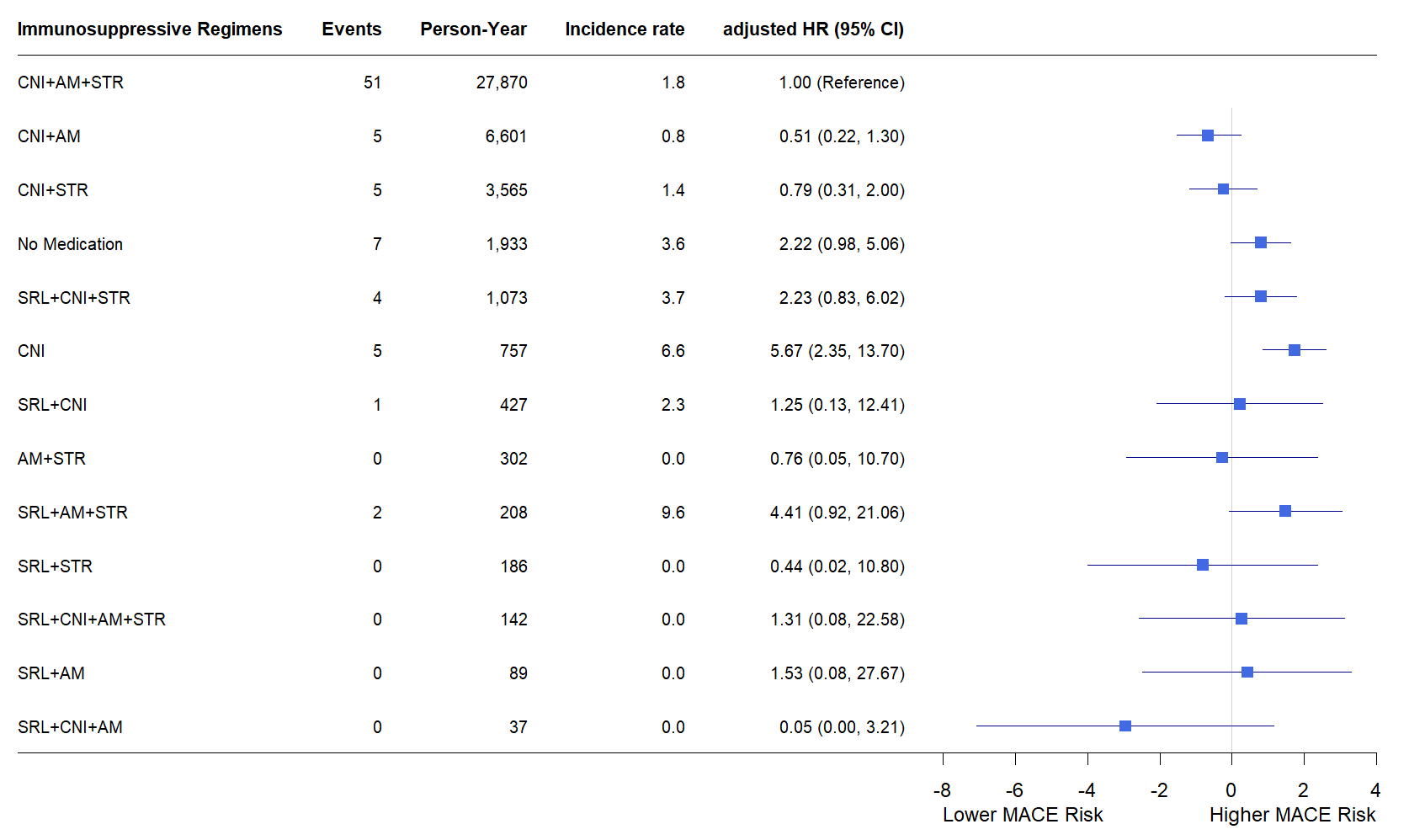


(B)


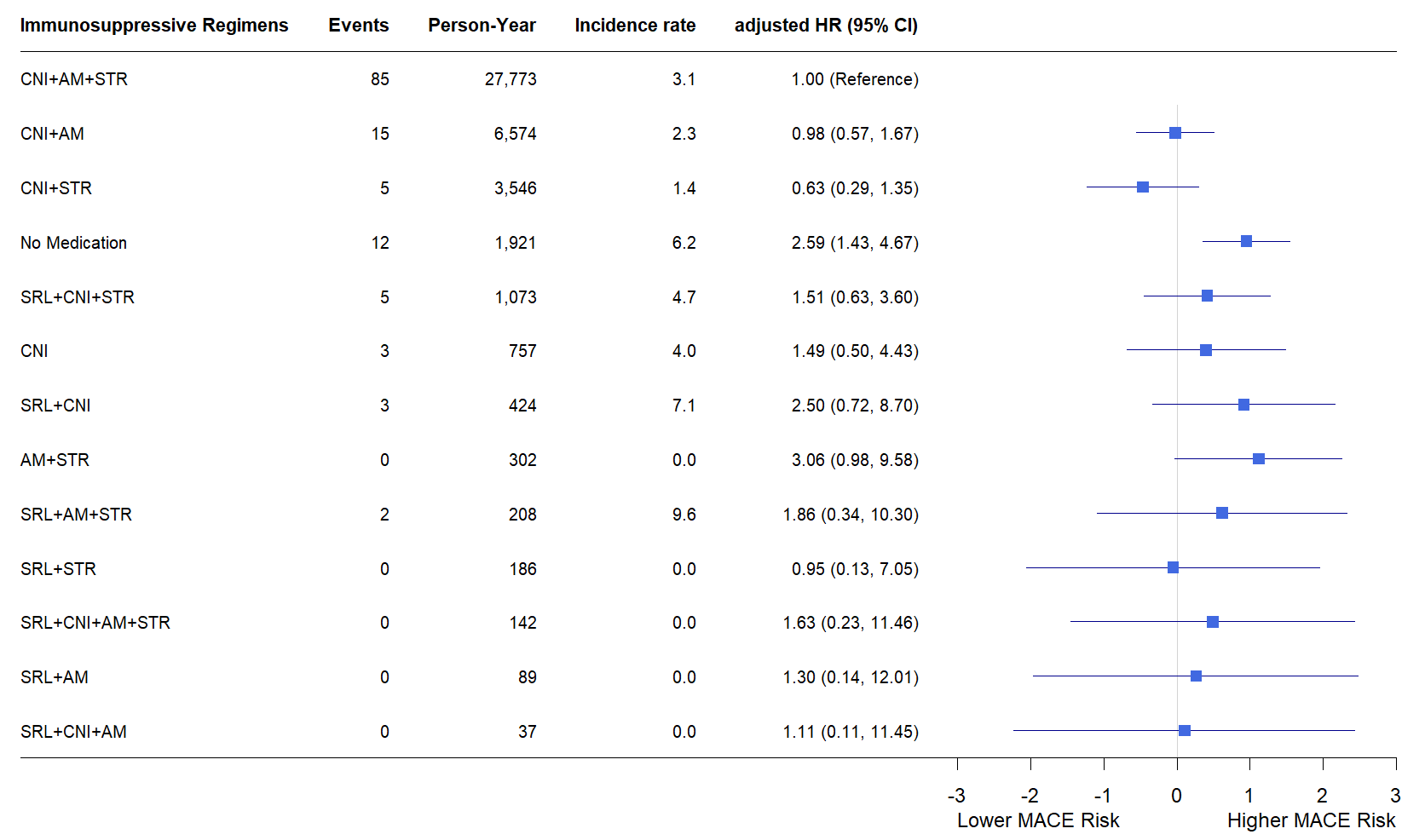


(C)


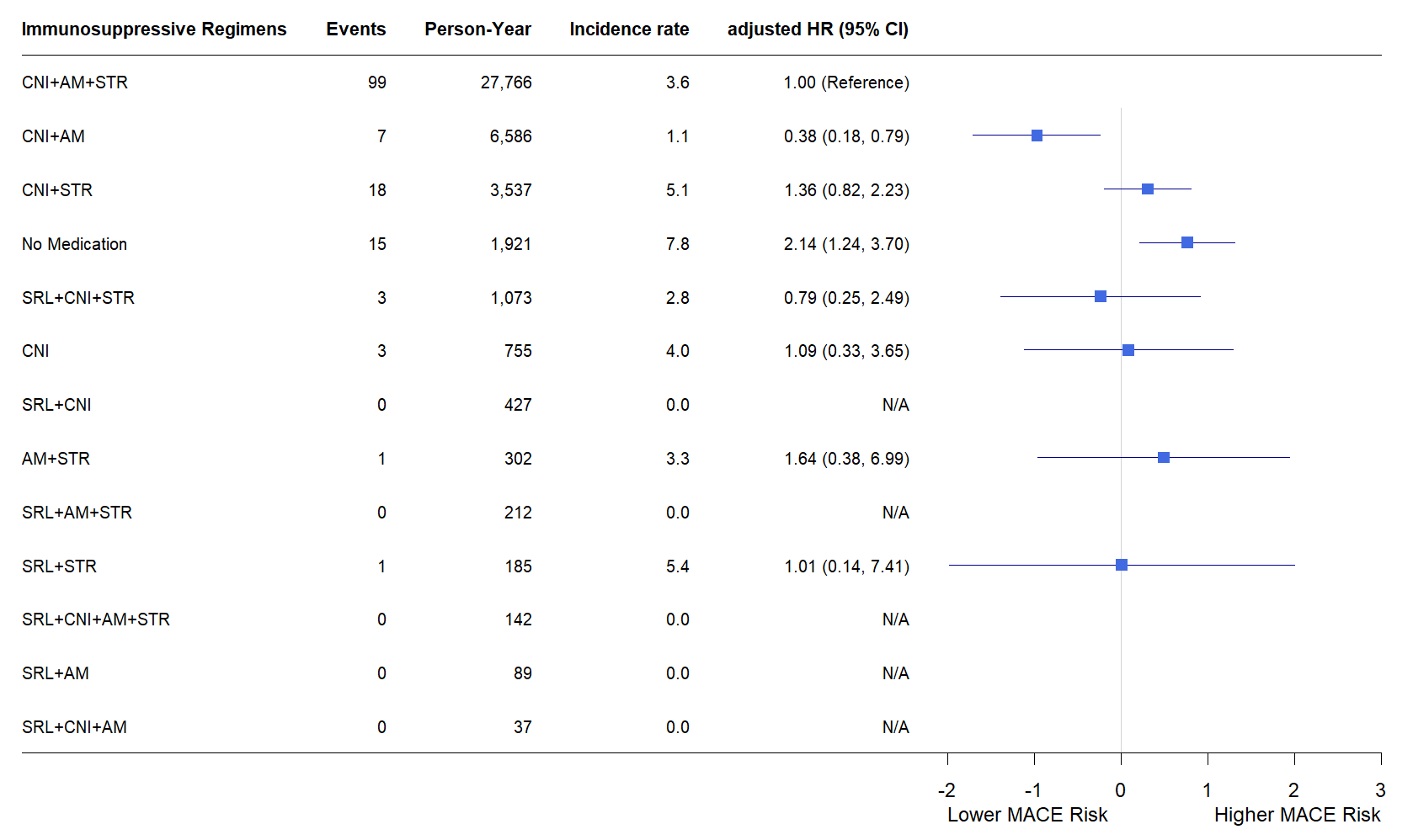


(D)


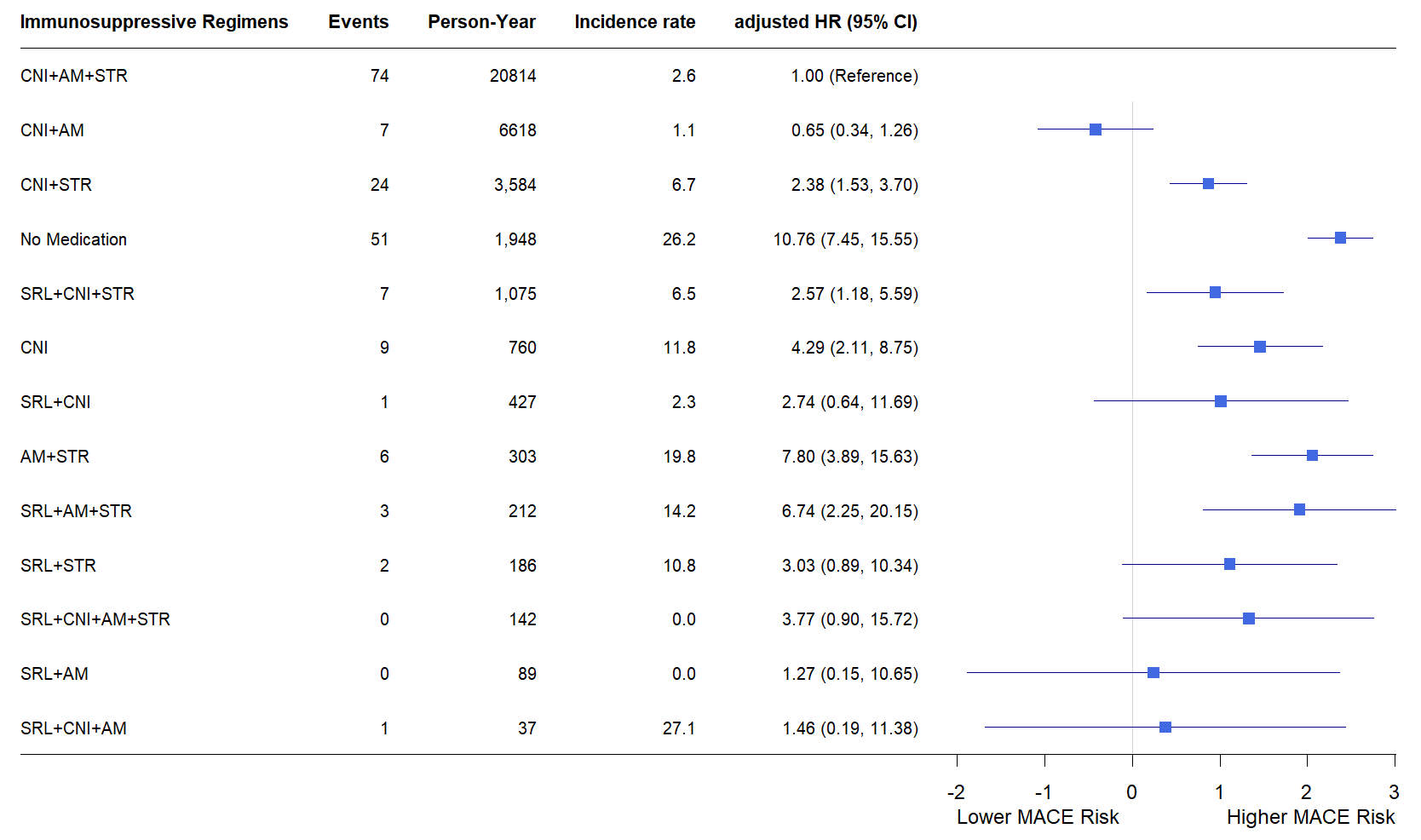


(E)


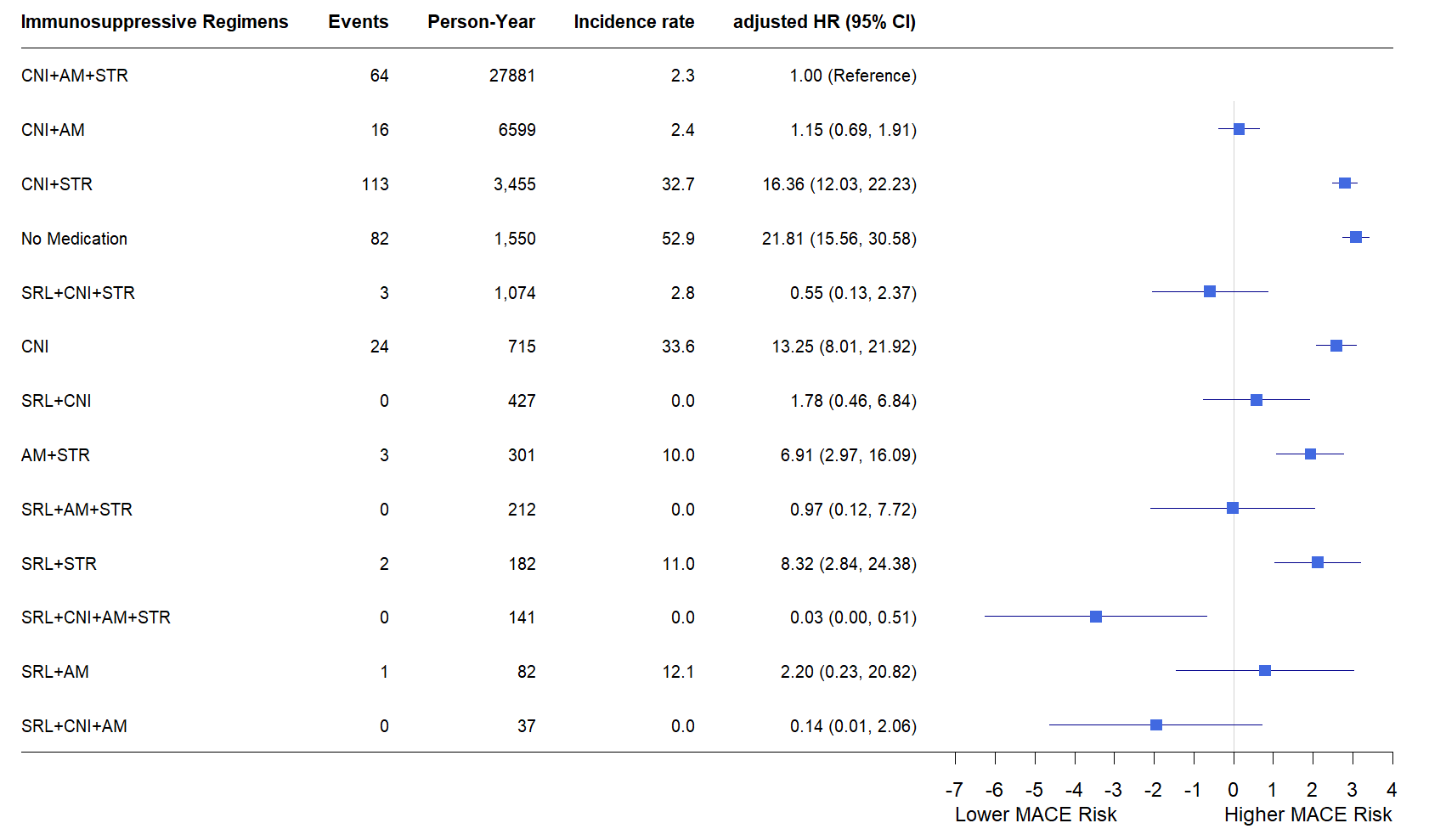


**Supplementary Figure 3 Incidence rate and hazard ratio of (A) myocardial infarction, (B) coronary revascularization, (C) ischemic stroke, (D) all-cause death, and (E) death censored graft failure according to immunosuppressive regimens.**

Each immunosuppressive regimen is represented by four types of immunosuppressive medication (SRL, CNI, AM, STR). incidence rates are expressed as the number of events per 1,000 person-years. Hazard ratios were adjusted for sex, age, transplant year, type of insurance, dialysis duration, donor type, presence or absence of desensitization, drugs used in Induction Therapy, CCI, underlying disease. Forest plot illustrating hazard ratios for major adverse cardiovascular events. The x-axis is presented on a natural log scale. Horizontal lines represent the 95% confidence intervals.

Abbreviations: SRL, sirolimus; CNI, calcineurin inhibitor; AM, antimetabolite; STR, corticosteroid; PY, person-year; IR, incidence rate; HR, hazard ratio; CI, confidence interval; N/A, not applicable.
